# Segmental colitis associated with diverticulosis: Progress to ulcerative colitis

**DOI:** 10.1002/deo2.379

**Published:** 2024-06-14

**Authors:** Akira Hokama

**Affiliations:** ^1^ Department of Medical Checkup Naha City Hospital Okinawa Japan

To the Editor,

I read with great interest the article by Obata et al.,^1^ who clearly described endoscopic and clinicopathological features of segmental colitis associated with diverticulosis (SCAD). I congratulate them on their efforts in studying the first Japanese case series of SCAD. I fully agree with their conclusion that aggressive anti‐inflammatory treatment should be considered for SCAD with ulcerative colitis (UC)‐like findings due to the potential risk of severe ulceration, stenosis, and/or perforation. In their study, the progression of SCAD to UC was not observed. I would like to share my short review regarding the progression of SCAD to UC.^2^ More than 30 cases of such progression have been reported to date, and among them, I have reviewed eight cases that were described in detail in the English literature. The review has shown that severe forms of SCAD may have the potential to progress to UC, which also have aggressive disease behavior requiring colectomy. A recent 3‐year prospective international cohort also revealed that 2.3% of patients with SCAD type B developed UC,^3^ suggesting a certain pathogenic relationship between the two diseases. Numerous factors, including smoking history and intestinal microbiota alteration, may influence this onset.^3^, ^4^ Further studies are needed to unveil this pathogenesis.
